# Comparative analysis of nutritional quality of five different cultivars of sweet potatoes (*Ipomea batatas* (L) Lam) in Sri Lanka

**DOI:** 10.1002/fsn3.38

**Published:** 2013-05-28

**Authors:** Suraji A Senanayake, K K D S Ranaweera, Anil Gunaratne, Arthur Bamunuarachchi

**Affiliations:** 1 Department of Food Science and Technology, Faculty of Applied Sciences, University of Sri Jayewardenepura, GangodawilaNugegoda, Sri Lanka; 2 Department of Agriculture, University of SabaragamuwaBelihuloya, Sri Lanka

**Keywords:** Amylose, digestibility, granular size, starch, sweet potato

## Abstract

Nutritional attributes of flours obtained from five different cultivars of sweet potato roots commonly available in Sri Lanka showed significant differences in the tested parameters. The starch level ranged between 33% and 64% on the dry basis and the extractability from fresh tubers was governed by the quantity of starch. The crude fiber level ranged between 2.1% and 13.6% on dry basis and the highest level was observed in swp7 (CARI 273) and resistant starch ranged from 14.2% to 17.2%. Higher percentage of resistant starch from total starch was found in Wariyapola red (swp1) cultivar resulting in lower digestion level while higher levels of digestion was evident in cultivars with lower levels of resistant starch with high level of total starch. Low levels of calcium and significant levels of iron were found in the five cultivars studied. Crude protein level was in the range of 1.2–3.3% on dry basis and trypsin inhibitor activity level (TIA) was significantly different (*P* > 0.05) in the cultivars studied while heating resulted in a significantly high reduction in the TIA level than in unheated condition. Polygonal or round shaped starch granules were in the range of 16.8–23.5 μm and low level of starch digestion was shown in cultivars containing larger granules. Total amylose content lies in the range 15.4–19.6% and cultivars having higher percentage of amylose showed higher level of in vitro pancreatic digestion (Pallepola [swp4] and swp7). The starch digestibility of sweet potato flour was in the range of 36–55% and the highest digestion was observed in swp7. Orange fleshed cultivars (swp4 and swp7) were comparatively rich in nutrients and digestibility than the other three studied cultivars.

## Introduction

The sweet potato (*Ipomea batatas* (L) *Lam*) is a tuberous-rooted perennial plant belonging to the Convolvulaceae or morning glory family. This family includes about 45 genera and 1000 species, but only *Ipomea batatas* is of economic importance as food (Onwueme 1978). Sweet potatoes are the seventh most important food crop grown in around 111 countries (Horton 1988). Today, the main commercial producers of sweet potatoes are China, Indonesia, Vietnam, Japan, India, and Uganda. Sweet potato plant consists of climbing stems, branches, petioles with grooves on the upper surface, leaves spirally arranged on stems and roots, and a plant produces one to several tuberous roots at maturity, generally four to seven (Chandra and Thivari 1985; Bouwkamp & Hassam 1988).

In Sri Lanka, sweet potato is a traditional crop grown mainly in the wet and intermediate zones and nearly about 50,000 tones are produced annually (UN – FAO stats, 2009). All of this production is used for human consumption as high consumer preference for roots prevents the utilization of it as an animal feed because the production is insufficient. Sweet potatoes are considered as an easily cultivatable and manageable crop as it can be cultivated under less favorable conditions with minimum amount of agricultural inputs. In addition, the crop is well adapted to extreme environmental conditions, also it can be cultivated with other crops, and can obtain a considerable amount of harvest if managed properly. As a result of these favorable features, an increased attention was received by sweet potato as a valuable food crop in the recent past in human nutrition and also it was reported that sweet potato production is about 2.1 kg/ha per day (Scott et al. 2000).

Sweet potato is mainly consumed by low income people because it is one of the cheap substitutes for starchy staples such as rice, wheat, and potatoes (De Silva and Jayawardene 1989) and contains a considerable level of starch, soluble sugars, vitamins, minerals, and other nutrients. Fresh tubers of sweet potatoes are commonly eaten boiled or as a curry with other food ingredients by the Sri Lankan community. The main objective of this study was to analyze the nutritional quality with regard to macro- and micronutrients, antinutritional factors (trypsin inhibitor levels) and the digestibility of flour and starch with relation to amylose content and starch granular size in five commonly consumed sweet potato cultivars in Sri Lanka. Such a study may demonstrate potential uses within the food industry in composite food mixtures as a nutrient-rich replacement of more traditional forms of carbohydrates or in new product development. The cultivars assessed in this study were crossbreedings, mutants of different genotypes, and introduced varieties to Sri Lanka and commonly consumed throughout the country. Samples which were kept under similar conditions 2–3 days after harvesting have been randomly selected from three different locations in three regions of the country obtained for analysis.

## Materials and Methods

### Raw material

Matured tubers of sweet potatoes namely, SWP1 (Wariyapola red), SWP 3 (Wariyapola white), SWP 4 (Pallepola variety), SWP 5 (Malaysian variety), and SWP 7 (CARI 273) were randomly collected from Dhambulla, Horana, and Gokarella areas in Sri Lanka and prepared for analysis 2–3 days after harvesting.

### Separation of different fractions

#### Flour extraction

The tubers were washed, hand peeled, and trimmed to remove defective parts. Then the tubers were grated into thin chips (∼5 mm) and dried in an air convention oven at 40°C for 30 h up to 14% moisture. The dried chips were powdered using a laboratory scale grinder and sifted through a 300 μm sieve. The flour samples were sealed and packed in airtight containers for further analysis and percentage extractability from fresh tubers at 14% moisture for each sample was determined.

#### Starch separation

Starch separation was carried out according to the method described by Takeda et al. 1988 with slight modifications. Fresh tubers were washed, peeled, and diced. These dices were dipped in ice water containing 100 ppm sodium metabisulfite to minimize browning and was wet milled at low speed in a laboratory scale blender with 1:2 w/v of tap water for 2 min and filtered through a gauze cloth. Residue was repeatedly wet milled and filtered for thrice and suspension was kept overnight for settling of starch. The supernatant was decanted and the settled residue was further purified with repeated suspension in tap water (1:2 v/v) followed by the settling process for 3 h. The purified starch was dried at 35°C, sifted through a 300 μm sieve, sealed, and packed for analysis.

### Chemical composition analysis of flour

Protein (N × 6.25) and fibre were determined according to Association of Official Analytical Chemistry (AOAC 1980), 7.015 (13) and AOAC (1980), 7.065 (13) methods, respectively. Starch content was estimated by complete acid hydrolysis method (Kent Jones and Amore 1960). Flour sample of 2.5 g was suspended in a mixture of 200 mL of water and 20 mL of HCl acid. (Sp. gravity 1.125) The mixture was heated in a flask provided with a reflux condenser for 2.5 h. The contents were cooled and neutralized with NaOH (5 N). The volume was made to 250 mL and the sugar formed was determined as dextrose by Lane and Eynon reducing sugar estimation method. The dextrose multiplied by 0.9 was taken as starch. Resistance starch was determined by using Megazyme-resistant starch assay kit (Megazyme Ltd., Bray, Ireland).

Mineral elements (Ca and Fe) were determined by dry ashing method. The ash was dissolved in conc. HCl, filtered, and diluted to 50 mL with distilled water. Prepared solutions were analyzed with standards for elemental analysis using Atomic Absorption Spectrophotometer (GBC Avanta ver 1.33, Buckinghamshire, UK).

### Trypsin inhibitor activity

Trypsin inhibitor activity (TIA) levels were analyzed in unheated and heated flour samples heated at 100°C by using the method described by Smith et al. (1980). TIs were extracted from heated and unheated samples with 0.0045 mol/L NaOH. Extracts were diluted, reacted with BAPNA (Sigma – Aldrich, Miamisburg, OH), and trypsin from porcine pancreas (Sigma – Aldrich), BAPNA and the absorbance at 410 nm was recorded. Milligrams of trypsin inhibited per 1 g of dry sample were calculated.

### Digestibility of sweet potato flour

Flour and starch digestibility was measured based on the method described by Zhang et al. (2002). A sample of 500 mg was placed in a weighed centrifuge tube (Tarsons, Chennai, India, 50 mL) with addition of 15 mL phosphate buffer (0.15 mol/L, pH 6.5), 30 mg CaCl_2,_, 30 mg gelatin, and 30 mg pancreatin (Sigma Co., St. Louis, MO). The capped tubes were placed in a shaking water bath at 37°C with a sufficient speed to keep the flour in suspension for 12 h and the reaction was stopped with addition of 5 mL of 1% H_2_SO_4_. The suspension was centrifuged at 20,000 *g* for 10 min and the supernatant was decanted and the residue pellet was dispersed with 15 mL of 80% ethanol and re-centrifuged for 5 min. The supernatant was decanted and the tubes with the residue pellet were dried at 50°C for 6 h, then at 80°C to constant weight, cooled, and weighed. Starch digestibility was expressed as percent weight loss after digestion. A blank without pancreatin was included for each sample to adjust the results.

### Total amylose content of the starch

Total amylose content of sweet potato starch was determined using spectrophotometric method after removal of lipids from starch with hot 75% *n*-propanol for 7 h in a Soxhlet extractor (Hoover and Ratnayake 2005). The pure potato amylose (Sigma – Aldrich) and amylopectin from maize (Sigma – Aldrich) were used to create a standard curve and the total amylose content of each sample was inferred from this standard curve. The difference was the considered as the amylopectin content.

### Determination of starch granular size

Granular size determination was carried out by Dinolite, versatile digital microscope (Magnification with mini tool box [FS] × 100) and the mean particle size diameter of granules appeared on a field was determined.

### Data analysis

Minitab software (Version 14) was used for Analysis of variance (ANOVA) with Tukey's honestly significant difference test (*P ≤* 0.05) to examine samples in triplicates for each parameter tested in different cultivars.

## Results and Discussion

### Nutrient composition

Flour extractability from fresh tubers lies in the range 40.2–55.1% at 14% moisture and starch level ranged between 33% and 64% on the dry basis (Table 1). The highest amount of starch was observed in swp7, while the lowest was seen in swp1 cultivar. The difference between the extracted starch and the total starch may be due to the presence of high level of soluble sugars and resistant starch (RS) present in the swp1 cultivar. The other four cultivars do not exhibit such a difference in the extractability and the total starch. Sweet potato contains about 25% dry matter and the exact amounts depend on the genetic variations, soil water level, physiological factors, and time, and conditions of storage. Dry matter content can range between 17.9% and 49.3% (Purcell et al. 1972). The mean total starch content within 44.4–74.5% in 108 genotypes was reported by Zhang et al. (2002) and the comparable results were reported by Bradbury et al. (1988). Total starch ranging from 30% to 85% on dry weight basis in different genotypes of sweet potatoes was reported by Bradbury and Holloway (1988).

**Table 1:** Macro- and micronutrient composition of flours obtained from sweet potato cultivars

Cultivar	Extractability of flour (%)	Total starch (g/100 g db)	Crude fibre (g/100 g db)	Iron (mg/100 g db)	Calcium (mg/100 g db)
Swp1	43.1 ± 0.7^c^	33.7 ± 1.7^e^	8.5 ± 0.4^b^	n.dt	n.dt
Swp3	51.0 ± 0.2^b^	58.6 ± 0.5^b^	7.3 ± 0.2^c^	6.2 ± 0.9^a^	2.1 ± 0.1^b^
Swp4	43.9 ± 0.2^c^	49.0 ± 0.3^c^	6.5 ± 0.4^d^	4.5 ± 0.9^b^	3.4 ± 0.8^b^
Swp5	40.2 ± 0.7^d^	43.0 ± 0.6^d^	2.1 ± 0.2^e^	4.2 ± 0.1^b^	5.9 ± 0.1^a^
Swp7	55.1 ± 0.1^a^	64.1 ± 1.9^a^	13.6 ± 0.3^a^	6.3 ± 0.2^a^	2.2 ± 0.1^b^

Data represent the mean of three replicates. Values followed by the similar superscripts in each column are not significantly different at (*P* > 0.05). n.dt, not determined.

The crude fibre level ranged between 2.1% and 13.6% on dry basis and the highest level was observed in swp7 and the lowest level seen in swp5. The level of crude fibre content in the roots was reported to range from 2.5% to 5.0% on dry basis (Jones et al. 1980). A dietary fibre level range from 5.25% to 7.14% was reported by Ahamed et al. (2010) at different drying temperatures of the flour. There is a significant difference (*P* > 0.5) in the crude fibre level in all the studied cultivars. Cellulose and lignin contents were gravimetrically measured in crude fibre determination and thus high level of RS in swp5 cultivar indicates the presence of high amount of dietary fibre in the particular cultivar.

Resistant starch level ranged from 14.2% to 17.2% on dry weight basis in the studied cultivars (Table 3). RS is significant as a functional food component as it is responsible for slow liberation and absorption of glucose in blood due to reduced digestion in the lower parts of the human gastrointestinal tract. This aids in the reduction in the risks of diabetes, obesity, and other related diseases (Liu et al. 2006). About half of the total starch content of swp1 cultivar is RS and all the cultivars contained a high level of RS which reduces the digestibility and thereby lowers the energy contribution. Although swp5 cultivar contained the highest level of RS, moderate level of digestion was observed (Fig. 3).

Iron and calcium contents range from 4.2 to 6.3 and 2.1 to 5.9 mg per 100 g dry weight, respectively. Calcium contents found in local cultivars are lower compared to the Malaysian cultivar and higher levels of calcium in sweet potato roots genotypes have been reported by Oke (1990) and Ishida et al. (2000) and comparably similar levels of Iron was found in studied cultivars. Very low levels of calcium and iron were reported in Nigerian varieties of sweet potatoes (Odebunmi et al. 2007).

### Protein content and TI levels

Crude protein content is in the range 1.2–3.3% on dry basis and protein levels range from 1.73% to 10.0% on dry basis as reported by researchers (Purcell et al. 1984; Bradbury et al. 1988). The highest level was observed in swp7 and the lowest found in swp5 (Table 2). The protein quality of sweet potato is of acceptable nutritive value in comparison with the FAO, 1973 reference and appreciable levels of amino acid lysine, which is deficient in rice are also found in sweet potato roots (Woolfe 1987). Although Purcell et al. (1972) reported the high levels of essential amino acids in sweet potatoes that are in the acceptable ranges of FAO reference, studies revealed a great deal of decrease in the amino acid content in heat-processed roots (Walter and Purcell 1986).

**Table 2:** Protein content, trypsin inhibitor activity (TIA), and digestibility of flours obtained from different cultivars of sweet potatoes

Cultivar	% Protein^1^ (db)	TIA (unheated) per/g sample (db)	TIA (heated) per/g sample (db)
Swp1	1.2 ± 0.1^e^	1.9 ± 0.01 ^h^	0.156 ± 0.01^j^
Swp3	3.0 ± 0.1^b^	6.2 ± 0.02^d^	3.03 ± 0.03^f^
Swp4	2.3 ± 0.1^c^	15.4 ± 0.4^a^	13.0 ± 0.01^b^
Swp5	1.7 ± 0.2^d^	3.8 ± 0.1^e^	1.56 ± 0.01^i^
Swp7	3.3 ± 0.1^a^	7.0 ± 0.1^c^	2.87 ± 0.01^g^

Data represent the mean of three replicates. Values followed by the different superscripts in each column are significantly different at (*P* < 0.05).

1 6.25 × N.

A wide variation in TIA levels of different sweet potato cultivars was observed (Table 2). The range of TIA in unheated flour samples of roots was from 1.9 ± 0.01 to 15.4 ± 0.4 and in the heated sample TIA value lies in the 0.156 ± 0.01 and 13.0 ± 0.01 range. Results clearly show a significant reduction IN TIA in heated samples compared to the unheated samples. A major antinutritional factor of sweet potato is the presence of high level of proteinase inhibitors. This group of protease inhibitors suppresses the action of proteinases and makes proteins nutritionally unavailable. The presence of TI in sweet potato was first reported by Sohonnie and Bhandarker (1954). Trypsin inhibitors in sweet potato storage roots account for about 60% of the total water soluble proteins and could be recognized as storage proteins (Lin 1989). It was reported that uncooked sweet potato roots in animal feed reduced the growth rate of pigs (Bouwkamp et al. 1988). Poor protein digestibility due to TI present in sweet potato might be a constraint in promoting the use of sweet potato as an animal feed (Zhang et al. 2002). Levels of TIA in sweet potato cultivars have been reported (Bradbury et al. 1988; Ravindran et al. 1995).

Results also revealed that heating the samples at 100°C for 5 min has reduced the TIA at *P* > 0.05% level significantly, though considerable levels still remain in heated samples. Lin and Tsu (1987) discovered that the heating at 100°C for 15 min completely inactivated the original TIA in sweet potato root flour samples. Inactivation of TI by heat treatment may improve the protein quality and thereby increase the nutritive quality of the food stuff. Swp3, Swp5, and Swp 7 cultivars contained fairly low levels of TI in unheated form and it is even lower in heated samples. High level of TIA in Swp4 may be due to different types of TI and/or due to variations in environmental factors.

### Starch granular size

**Figure 1:**
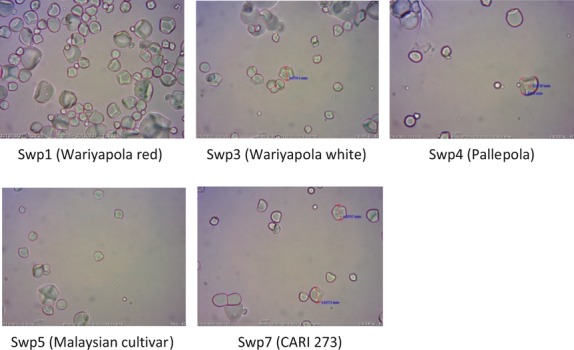
Light microscopic structure of starch granules from different cultivars (10 × 100).

**Figure 2:**
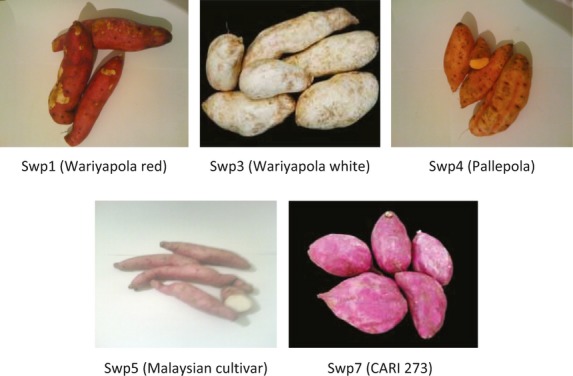
Different cultivars of sweet potatoes.

Starch granules appeared as polygonal or round in shape (Figs. 1 and 2) and the size varied in the range 16.8–23.5 μm. A wide range of 2–80 μm was reported by Swinkels (1985). Wickramasinghe et al. (2009) reported 12–19.4 μm range of granular size in three different cultivars of sweet potatoes from Sri Lanka and Japan.

Moorthy (2002) found a strong positive correlation between the starch granular size with the amylose content: larger the starch granule, higher was the contents of amylose found. This relationship was not evident in the studied cultivars (Table 3). Bouwkamp et al. (1988) reported a negative correlation between the granular size and the digestibility by α-amylase in different cultivars of sweet potatoes. This was evident in swp5 with lower digestion with the highest granular size while swp4 and swp7 having higher percentage of digestion with relatively smaller sized starch granules.

**Table 3:** Content of total amylose, amylopectin, resistant starch, and starch granular size of starches obtained from different sweet potato cultivars

Cultivar	Amylose (%)	Amylopectin (%)	Resistant starch (%)	Granular size (μm)
Swp1	15.4 ± 0.4^e^	84.6 ± 0.4^a^	14.2	17.0 ± 0.2^c^
Swp3	17.7 ± 0.2^c^	82.3 ± 0.2^c^	15.1	16.8 ± 0.1^c^
Swp4	18.6 ± 0.1^b^	81.4 ± 0.1^d^	14.8	18.1 ± 0.4^b^
Swp5	16.6 ± 0.1^d^	83.4 ± 0.1^b^	17.2	23.5 ± 0.1^a^
Swp7	19.6 ± 0.1^a^	80.4 ± 0.1^e^	13.2	18.5 ± 0.3^b^

Data represent the mean of three replicates. Values followed by the different superscripts in each column are significantly different at (*P* > 0.05).

### Total amylose content

Total amylose content lies in the range 15.4–19.6% and is significantly different (*P* > 0.05) in all the cultivars studied. Highest and lowest levels were reported in swp7 and swp1, respectively. Total amylose content ranging from 14.9% to 25.1% in 114 genotypes of sweet potatoes was reported by Zhang et al. (2002) and also there was no significant difference found in the amylose content with the varying environmental factors. Wickramasinghe et al. (2009) reported 16.6% and 19.3% of apparent amylose contents in two different cultivars of Sri Lankan sweet potatoes. Amylose content ranging from 8.5% to 38% was reported by Rasper (1969). Tsou and Hong (1989) reported that starch characteristics such as higher amylose content, lower phosphorous content, and higher gelatinization temperature could be associated with greater susceptibility to in vitro digestion. This factor was evident in our study as cultivars having higher percentage of amylose showing higher level of in vitro pancreatic digestion (swp4, swp7). And also swp1 with the lowest amylose% showing the lowest level of digestibility with comparably similar level of digestion with similarities in percentage amylose content (swp3, swp5).

### Digestibility of sweet potato flour

The starch digestibility of sweet potato flour was in the range 36–55% and the lowest and highest digestions were observed in swp1 and swp7, respectively. The presence of low level of total starch together with high levels of crude fibre and RS may have caused the poor digestibility in swp1 cultivar. On the other hand, swp7 shows a high level of digestibility with significantly high level of crude fibre and RS. The digestibility range of sweet potato starch of 108 studied genotypes by Zhang et al. (2002) is in the range 29.5–63.6% and a similar range was found also by Zhang et al. (1993) and Zhang and Oates (1999). Our results were comparable to previous findings.

Variation in starch digestibility of sweet potato flour may be due to the different properties of the raw starch. The digestibility of raw starch by α-amylase is influenced by factors such as amylose content (Sugimoto et al. 1982) or starch granular size (Noda et al. 1996). It was also evident that the digestibility of starch and flour of sweet potatoes was similar as starch is the main component in flour thus, pancreatic α-amylase determines the extent of hydrolysis of flour (Zhang et al. 1993).

**Figure 3:**
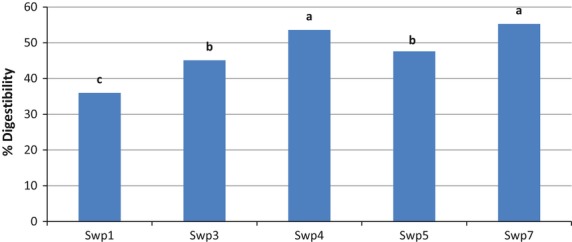
Percentage pancreatic digestibility of flours from different cultivars of sweet potatoes. Bars labelled with similar letters are not significantly different at *P* > 0.05 level.

Correlation studies revealed a high level of digestibility of genotypes with higher dry matter and starch contents (Zhang et al. 2002). This is evident in swp7 cultivar with high level of starch than the other cultivars and lower digestibility of swp1 with low content of total starch (Fig. 3). No significant level of variation in digestion was found in cultivars (swp4, swp7) with similar starch granular size (*P* > 0.05) and digestion level was similar though there was a significant difference (*P* > 0.05) in the granular sizes (swp3, swp5). The degree of digestibility depends on the chemical nature of the starch, physical form, the presence of possible inhibitors, and the physical distribution of starch with relation to dietary fibre components such as cellulose, hemicelluloses, and lignin (Snow and O'Dea 1981). Apart from the high level of crude fibre and amylose contents in cultivars swp4 and swp7 the high level of digestibility in them may be due to the chemical nature, comparatively low level of RS and the absence of possible inhibitors in the flours obtained from those cultivars.

## Conclusions

Flours and starches separated from commercially available different cultivars of sweet potatoes in Sri Lanka showed obvious differences in macro- and micronutrients, TI levels, and total amylose content. There was no significant difference in the digestibility level and granular size of certain cultivars at *P* > 0.05 level (swp3, swp5 and swp4 and swp7). As a whole, from the tested parameters, swp7 can be considered as the most nutritionally significant cultivar than the four others tested with regard to nutrient richness and digestibility. However, for a more conclusive study it is suggested to analyze these cultivars obtained from different locations of this country during varied months of the year.
